# Hierarchical Aerographite 3D flexible networks hybridized by InP micro/nanostructures for strain sensor applications

**DOI:** 10.1038/s41598-018-32005-0

**Published:** 2018-09-17

**Authors:** Irina Plesco, Julian Strobel, Fabian Schütt, Cameliu Himcinschi, Nabiha Ben Sedrine, Teresa Monteiro, Maria Rosário Correia, Leonid Gorceac, Boris Cinic, Veaceslav Ursaki, Janik Marx, Bodo Fiedler, Yogendra Kumar Mishra, Lorenz Kienle, Rainer Adelung, Ion Tiginyanu

**Affiliations:** 1 National Center for Materials Study and Testing, Technical University of Moldova, Stefan cel Mare av. 168, MD-2004 Chisinau, Republic of Moldova; 2 Institute for Materials Science, Kiel University, Kaiserstr. 2, D-24143 Kiel, Germany; 3 Institute of Theoretical Physics, TU Bergakademie Freiberg, Leipziger Str. 23, D-09596 Freiberg, Germany; 4 Department of Physics and I3N, Institute for Nanostructures, Nanomodelling and Nanofabrication, University of Aveiro, P-3810-193 Aveiro, Portugal; 5 Department of Physics and Engineering, State University of Moldova, Alexei Mateevici str. 60, MD-2009 Chisinau, Republic of Moldova; 6 Institute of Polymers and Composites, Hamburg University of Technology, Denickestr. 15, D-21073 Hamburg, Germany

## Abstract

In the present work, we report on development of three-dimensional flexible architectures consisting of an extremely porous three-dimensional Aerographite (AG) backbone decorated by InP micro/nanocrystallites grown by a single step hydride vapor phase epitaxy process. The systematic investigation of the hybrid materials by scanning electron microscopy demonstrates a rather uniform spatial distribution of InP crystallites without agglomeration on the surface of Aerographite microtubular structures. X-ray diffraction, transmission electron microscopy and Raman scattering analysis demonstrate that InP crystallites grown on bare Aerographite are of zincblende structure, while a preliminary functionalization of the Aerographite backbone with Au nanodots promotes the formation of crystalline In_2_O_3_ nanowires as well as gold-indium oxide core-shell nanostructures. The electromechanical properties of the hybrid AG-InP composite material are shown to be better than those of previously reported bare AG and AG-GaN networks. Robustness, elastic behavior and excellent translation of the mechanical deformation to variations in electrical conductivity highlight the prospects of AG-InP applications in tactile/strain sensors and other device structures related to flexible electronics.

## Introduction

Over the last few years, increasing attention has been paid to the development of flexible nanocomposite hybrid materials based on carbon aerogels decorated by semiconductor nanoparticles as next-generation nanomaterials for electronic, photonic and sensor applications. Carbon foams such as graphene aerogels (GA)^1^ and Aerographite (AG)^2^ represent promising scaffolds for the deposition of various solid-state nanoparticles, resulting in the formation of hybrid nanocomposite materials with flexible three-dimensional (3D) architectures. Recently 3D architectures of GaN and ZnO micro/nanocrystallites deposited on AG scaffolds have been proposed for micro- and nano-optoelectronic applications^3,4^, in particular for the development of flexible broadband photodetectors covering the spectral range from ultraviolet to infrared^4^. Linking nanoparticles to highly porous 3D skeleton of GA or AG via chemical bonds might be very important for biomedical applications since it avoids nanoparticle agglomeration occurring in liquids^3^. When nanoparticles of electrostrictive semiconductors are deposited on GA/AG templates in the form of continuous nanocrystalline films, the resulting nanocomposites represent materials of choice for the fabrication of ultra-lightweight flexible pressure sensors, which are promising for applications in automotive and aeronautic industries^5^.

Flexible materials based on Aerographite decorated by semiconductor nanoparticles exhibit electromechanical characteristics which hint to the possibility to use them in tactile/strain sensors. The results of previous investigations of the AG-GaN nanomaterial^3^ show, however, that the fabrication of AG-based hybrid networks with as high robustness and elasticity as to assure a reliable and repeatable modulation of the electrical conductivity under compression and decompression is challenging.

Indium phosphide (InP) is a binary semiconductor compound with the direct bandgap of 1.28 eV (at 300 K), which is broadly used in high-frequency electronics^6–9^, while InP-based light emitters are used in optical telecommunication systems and biomedicine^6,10,11^. Taking into account the high radiation hardness of InP, considerable efforts have been undertaken over the last decade to produce InP solar cells for use in space^12^. Besides, nanoporous membranes of InP were found to exhibit artificial birefringence, enhanced optical second harmonic generation as well as enhanced terahertz emission under excitation by near-IR femtosecond laser pulses^13–15^. In this paper, the fabrication and systematic characterization of the morphology, crystallinity, compressibility and optical properties of 3D flexible architectures consisting of Aerographite decorated by InP micro/nanoparticles is reported. Besides, the influence of preliminary functionalization of the AG template by Au dots on the morphology and crystallinity of the deposited InP particles is studied and the formation of In_2_O_3_ nanowires and Au/In_2_O_3_ core-shell structures is disclosed. The advantages of AG-InP hybrid composite material in comparison with a previously reported AG-GaN structure^3^ for tactile/strain sensor applications are discussed.

## Results and Discussion

### Growth of InP on pristine Aerographite

**Figure 1:**
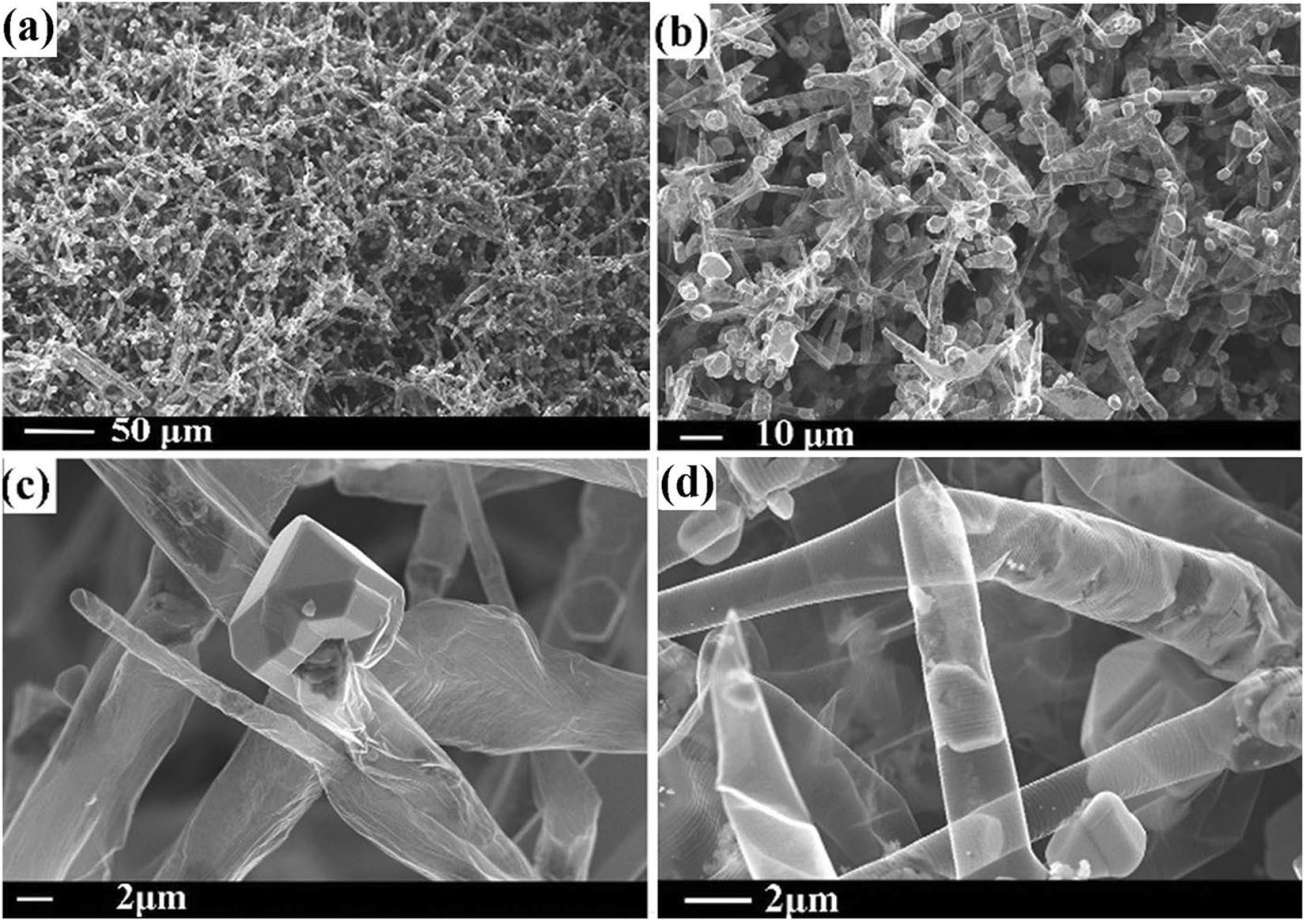
SEM images taken from AG decorated by InP microcrystallites: (**a**) and (**b**) large area views of the distribution of InP microcrystals on the Aerographite scaffold, (**c**) a high magnification SEM image of an InP microcrystal grown on the outer surface of the Aerographite microtube, (**d**) a high magnification SEM image of an InP microcrystal grown inside the Aerographite microtube.

The process of epitaxial growth of InP:Zn crystals on the Aerographite substrate was performed using hydride vapor phase epitaxy (HVPE) technology in the system In-PCl_3_-H_2_^16^. According to the results of our systematic investigations, nucleation and growth of InP nanocrystallites start at various nucleation points on the outer surface of AG tubular structures. With ongoing InP deposition, the InP nanocrystallites grow in size (Fig. 1a,b) and adjacent particles merge together to form microcrystallites (Fig. 1c). The distribution of InP microcrystallites along the outer surface of the AG network is relatively uniform, although their dimensions vary from a few nanometers to micrometer scales (Fig. 1b). It has to be noted that the microcrystallites exhibit well-developed facets thus reflecting the underlying symmetry of the *crystal structure* (Fig. 1c). The selected area electron diffraction (SAED) pattern of an InP microcrystal presented in Fig. S1 in the Supplementary Information demonstrates the single crystalline (space group ${\rm{F}}\bar{4}3{\rm{m}}$) structure.

Nucleation and formation of InP microcrystallites is also observed on the inner surface of AG microtubes (Fig. 1d). The growth of InP on the inner surface can be attributed to the penetration of HVPE reactants into the hollow microtubes via their openings or through pores in the walls of AG microtubes. However, one should note that, although the InP nanoparticles grown inside of AG microtubes sometimes exhibit facets, in most cases the microcrystals are not terminated by defined crystallographic planes (Fig. 1d).

**Figure 2:**
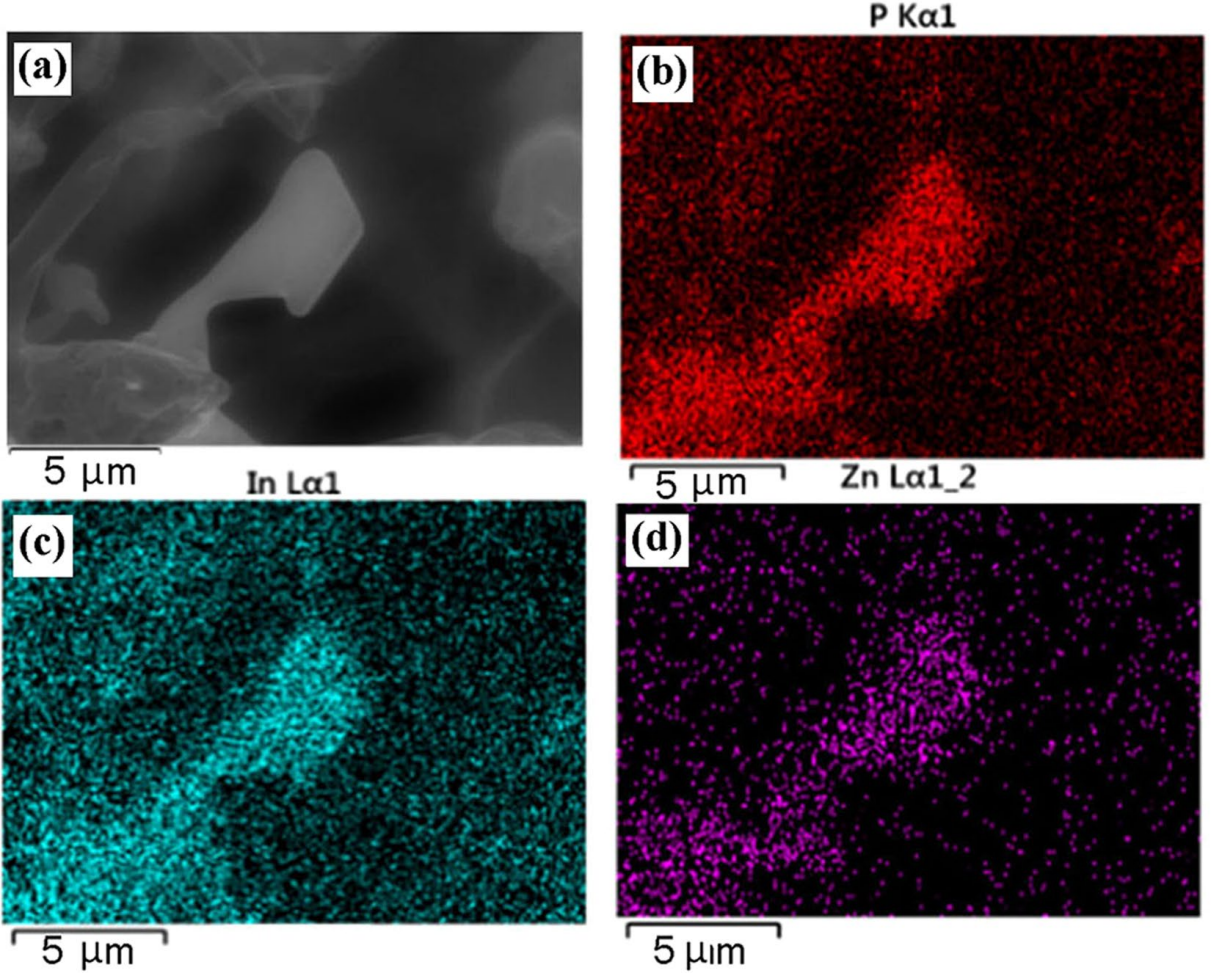
SEM image (**a**) and spatial distribution of chemical components across an InP microcrystal measured with EDX facilities (**b,c,d**).

The InP crystallites have been doped by Zn impurity for enabling p-type conductivity. The energy dispersive X-ray spectroscopy (EDX) mapping analysis of an InP microcrystal demonstrates a uniform distribution of the In and P components as well as of the doped Zn impurity (Fig. 2). One may suggest that some of the Zn atoms might stem from the incomplete reduction of ZnO during the AG formation. However, one should mention that no ZnO was detectable in the bare AG template, while the presence of Zn is detectable and confined in the InP structures.

**Figure 3:**
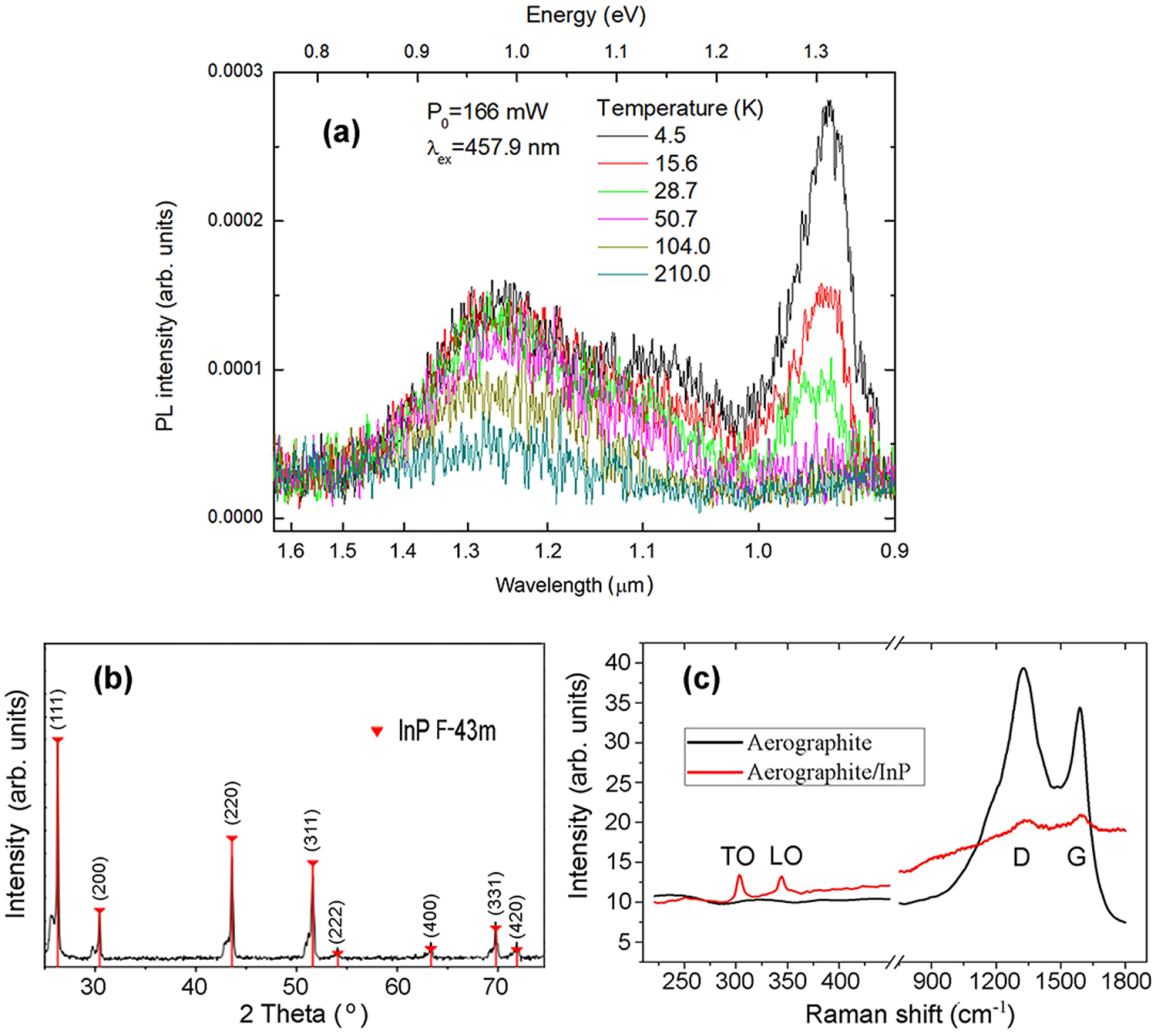
(**a**) PL spectra of InP crystallites measured at different temperatures. (**b**) XRD pattern of the InP crystallites. The triangles mark the peaks from the PDF 32-452 card. (**c**) Raman scattering spectra taken from Aerographite before and after functionalization by InP micro/nanocrystallites.

The impact of doping with Zn impurity upon the radiative properties and possible formation of point defects is disclosed by the analysis of photoluminescence (PL) spectra presented in Fig. 3a. The PL spectrum of the InP crystallites measured at low temperatures consists of an emission band at ~1.31 eV, i.e., in the near-bandgap region, and a deep emission band at around 0.97 eV. The emission at ~1.31 eV slightly redshifts with increasing temperature, and vanishes around 50 K, while the deep emission persists up to temperatures around 200 K. Two PL bands were previously found at 1.30 eV and 0.91 eV in InP crystals doped with Mg by means of ion implantation which were attributed to radiative recombination of non-equilibrium carriers via complex defect centers involving Mg_In_ and Mg_i_ point defects^17^. Since InP crystallites deposited on the Aerographite scaffold are doped by Zn acceptor impurity, one can suggest that the PL bands at 1.31 eV and 0.97 eV are generated by the radiative recombination of carriers via complex defect centers involving Zn_In_ and Zn_i_ point defects. The absence of excitonic emission in the PL spectrum is caused by the high concentration of Zn related defects. Note that, although InP is a direct bandgap semiconductor, direct band-to-band transitions are also absent in the PL spectrum since the defect-related recombination channels are not saturated at the applied low excitation power densities.

The X-ray diffraction (XRD) pattern of the InP crystallites reflects their ${\rm{F}}\bar{4}3{\rm{m}}$ zincblende structure (Fig. 3b). The growth of InP crystallites with the zincblende structure is also corroborated by the results of Raman scattering (RS) investigations (Fig. 3c). The RS spectrum of the Aerographite investigated using 633 nm excitation wavelength and 0.6 mW laser power shows typical C-C vibrational modes at 1327 and 1590 cm^−1^ assigned as D and G bands, respectively^18^. As expected, the spectrum measured in an area with the laser beam focused on an InP crystallite shows strongly suppressed D and G bands, comparing to the spectrum taken from pure Aerographite, and the predominance of the TO (303 cm^−1^) and LO (344 cm^−1^) modes inherent to zincblende InP structure. The spectral positions of vibrational modes in the InP microcrystal match well with those of LO and TO modes in the bulk material^19,20^. It was found that with increasing the excitation power density the RS bands of InP crystallites are broadened and redshifted due to the sample heating. At relatively high excitation densities, the spectrum undergoes strong and irreversible changes, with appearance of new features, which could be explained by the inter-diffusion of species at the InP/Aerographite interface^20^ and *in situ* InP oxidation^21^ due to strong laser induced heating, especially for the 633 nm resonant excitation (see Figs S2 and S3 in the Supplementary Information).

One can conclude that relatively large zincblende InP particles with dimensions of up to 10 µm grow on the outer and inner surfaces of microtubes constituting the AG network. The disclosed growth features are related to the specific properties of the AG template. Note that there should be no dangling bonds at the surface of an ideally perfect AG microtube, i.e., a crystallographically perfect AG microtube has no surface defects where the growth of InP crystallites could be initiated. However, real AG templates obviously contain lattice defects, which are responsible for the initiation of the growth of InP nanocrystallites. Taking into account the high diffusion velocities of In and P species along the surface of AG microtubes at the temperature of the HVPE process, one can expect that the In and P atoms easily reach the nucleation sites, which results in the growth of relatively large InP crystallites.

### Growth of micro/nanostructures on Aerographite functionalized by Au dots

**Figure 4:**
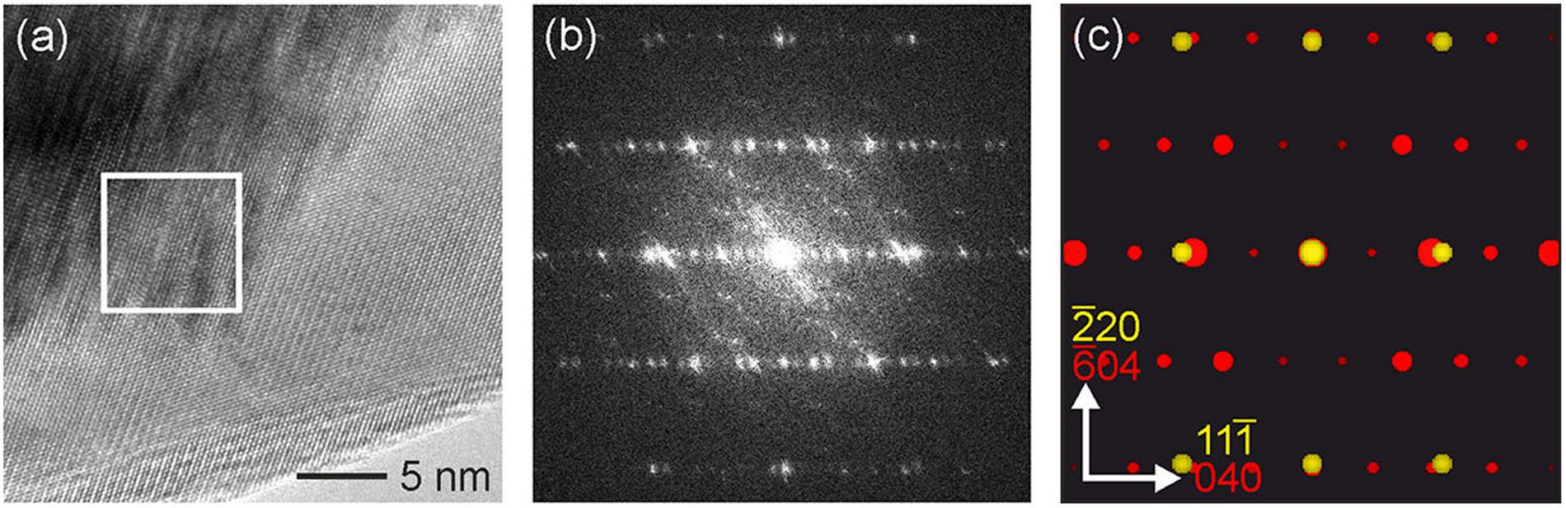
HRTEM investigation of Au/In_2_O_3_ core/shell particles. (**a**) HRTEM micrograph of such a particle showing both the fringe (bottom right) and the core (top left). (**b**) FFT of the region marked by a white square in (**a**). (**c**) Superposition of two simulated diffraction patterns of Au (yellow, ZA [112]) and In_2_O_3_ (red, ZA [203]) matching well with the FFT shown in (**b**). Note that (**b**) contains many additional reflections stemming from double diffraction.

A way for controlling the spatial distribution of semiconductor particles on the surface of the AG template is to preliminarily deposit an array of Au nanoparticles which acts as a catalyst in the semiconductor growth process (Fig. S4, Supplementary Information). At the first stage of the HVPE process, semiconductor nanoparticles start to grow at the deposited Au nanoparticles. The performed analysis shows that the shape of the produced particles and the mechanisms of growth in this case are different from those inherent to the pristine AG template without catalyst nanoparticles. Core/shell nanoparticles with a size of 20–100 nm are formed at the initial phase of the deposition process with Au catalyst (Fig. S4, Supplementary Information). The high resolution transmission electron microscopy (HRTEM) analysis suggests that these nanoparticles contain a heavy core and a light shell (Fig. 4). The fast Fourier transform (FFT) of the core and the shell regions of the nanoparticle demonstrates that Au (zone axis (ZA) [112]) constitutes the core, while the shell consists of indium oxide (In_2_O_3_, ZA [203]), apparently epitaxially intergrown (cf. Figure 4). This is also highlighted by the HRTEM, FFT and the SAED pattern analysis presented in Fig. S5 in the Supplementary Information.

**Figure 5:**
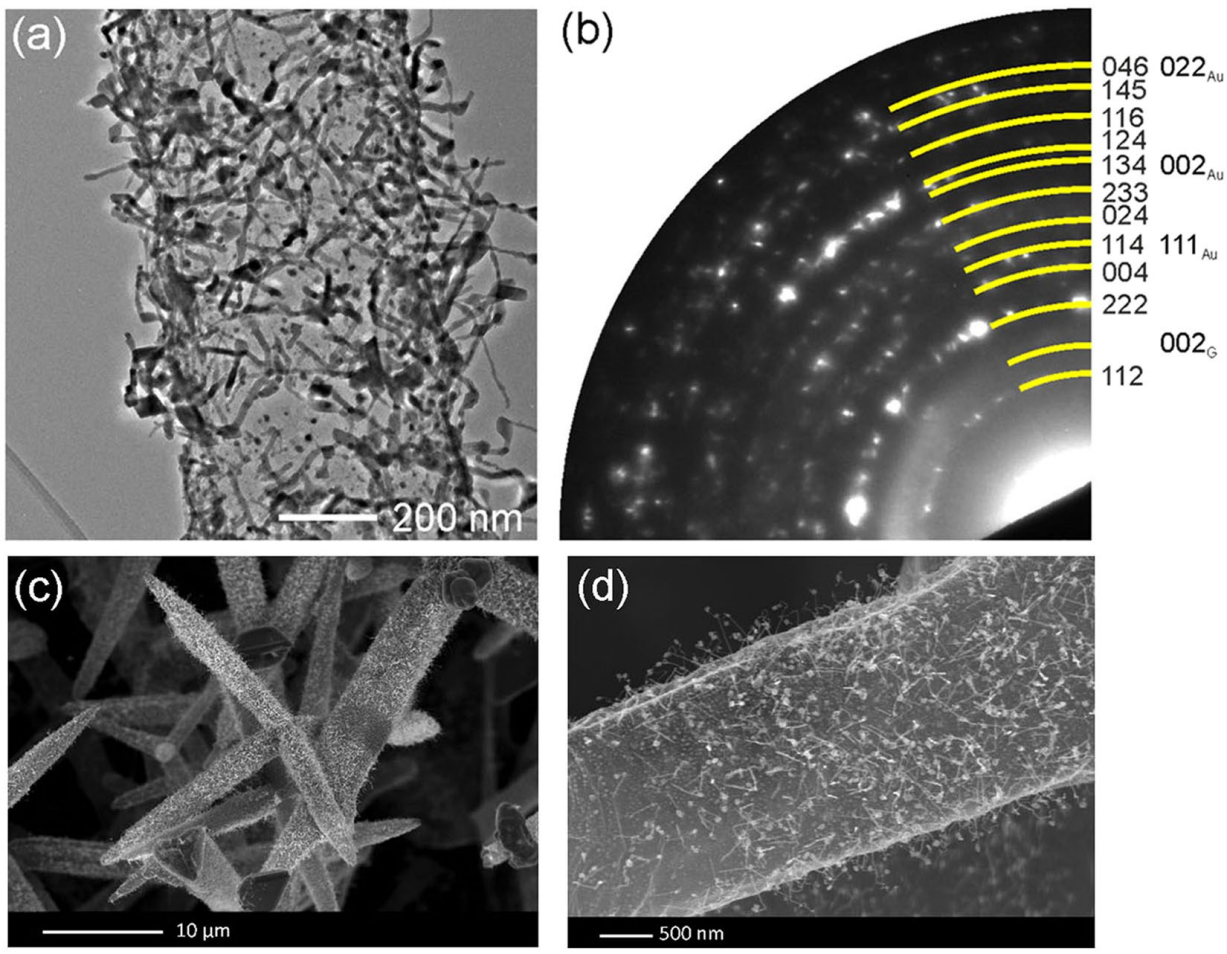
Morphology and structure of wire-like nanostructures deposited on AG. (**a**) TEM image of an AG microtube with deposited nanowires. (**b**) SAED pattern of the upper part of a nanowire. Yellow marks indicate diffraction maxima, indices to the right of the diffraction pattern belong to In_2_O_3_ (left column) and Au or graphite, respectively (right column). (**c,d**) SEM images of AG microtubes with deposited semiconductor nanowires.

**Figure 6:**
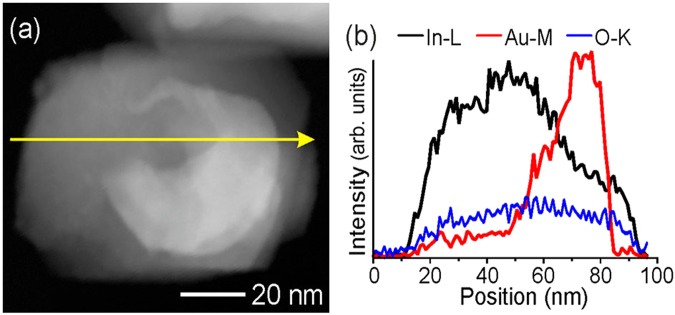
Elemental distribution in Au-In_2_O_3_ core-shell nanoparticles. (**a**) STEM-HAADF image of one core-shell particle. The yellow arrow marks the line where the line scan in (**b**) has been recorded. (**b**) EDX line scan recorded from the particle in (**a**). The bright core clearly correlates with the Au-M signal, while the shell contributes to In-L and O-K signals.

With the time of the HVPE process, nanowires start to grow on the AG microtube as shown in Fig. 5. Note that the growth of semiconductor nanowires with Au catalyst is inherent to vapor-liquid-solid processes^22–25^. Interestingly, the core/shell nanoparticle grown in the initial phase of the HVPE process, in most cases, is lifted-up by the growing nanowire. The SAED pattern taken from this structure conclusively shows that only In_2_O_3_ (sg. Ia3), Au and graphite phases are present. The HRTEM analysis of a nanoparticle with a nanowire attached to the AG template also demonstrates that both the nanowire and the nanoparticle shell are crystalline with the lattice spacing of 0.709 nm corresponding to the interplanar spacing of (011) In_2_O_3_ planes (see Fig. S6 in the Supplementary Information). This is corroborated by the results of EDX mapping, which confirm that the nanowire and the nanoparticle shell consist of In_2_O_3_, while the core of the nanoparticle is composed of gold (see Fig. 6 and Fig. S7 in the Supplementary Information).

As compared to bulk InP microcrystallites, the catalytically grown nanowires are composed of In_2_O_3_ rather than InP. Considering that no oxygen is involved in the HVPE growth process, one can assume that the initially grown nanowires consisted of InP. After growth, however, self-oxidation of InP nanowires is expected to occur under exposure to atmospheric oxygen. Indeed, it is well known that exposure of InP nanoparticles to air results in rapid oxidation^26^. It was pointed out that, while the fast oxidation of InP nanoparticles may be undesirable from the point of view of some applications, it provides a way for the fabrication of In_2_O_3_ nanostructures which themselves are of great interest^26^. In_2_O_3_ is an n-type, wide band-gap (~3.6 eV) semiconductor with wide prospects of implementation in solar cells, flat panel displays^27^ and nanoscale optoelectronic devices^28^. Particularly, In_2_O_3_ nanowires demonstrate enhanced photoelectrochemical performance for water splitting^24^ and enhanced gas-sensing properties^25^.

**Figure 7:**
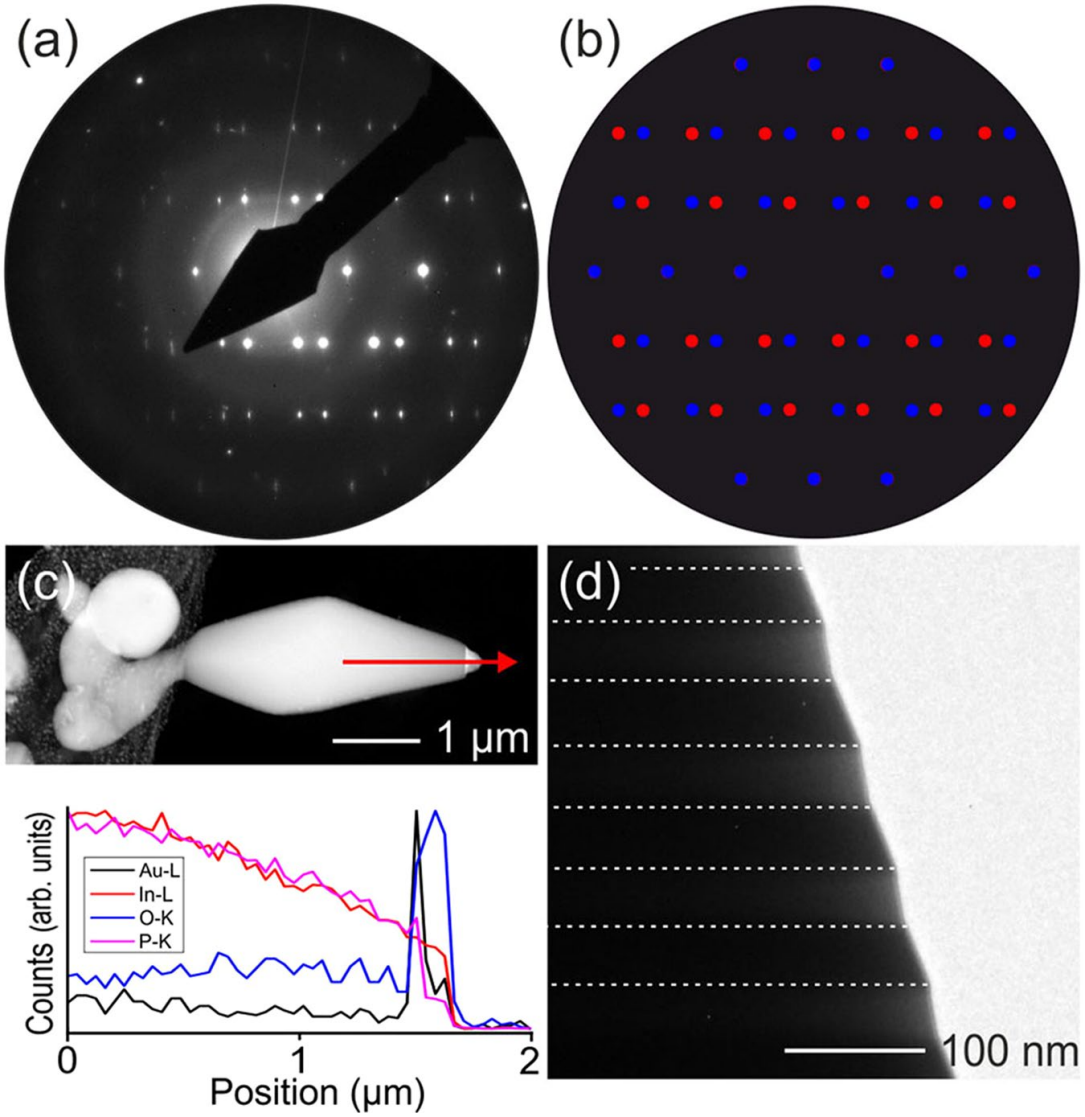
Analysis of InP microstructures. (**a**) SAED pattern of the InP micro-rod shown in (**c**). (**b**) Superposition of two simulated diffraction patterns of InP (ZA [1-10]) mirrored at the (112) plane, highlighting the twinned nature of the micro-rod. (**c**) Overview image and EDX line scan of the micro rod. The line scan has been recorded near the end of the rod, as indicated by the red arrow. The cap exhibits strong signal of Au as well as of O and In at its apex, indicating the Au/In_2_O_3_ composite cap. (**d**) Close-up of the micro rod. Dashed lines highlight the individual alternating twinning domains, apparent form their different contrast and slope near the edge. Unfortunately, the microrod was too thick for a detailed high resolution analysis of the twin boundaries.

It is noteworthy that relatively large arrow-shaped InP rods grow in some areas of the AG template (probably in areas containing prominent structural defects), as shown in Fig. 7c. The EDX analysis of such a microstructure demonstrates that it consists of a monolithic InP base with a cap of Au covered by an indium oxide shell (Fig. 7c). These observations corroborate the lift-up of the core/shell nanoparticles by the growing nanowires, as described above. The TEM analysis confirms that the InP rods, similarly to microparticles grown without Au catalyst, have a ${\rm{F}}\bar{4}3{\rm{m}}$ crystal structure. However, both the bright field image and the diffraction (Fig. 7a,b,d) show a twinned structure with (112) twinning plane, the individual domains are 25–35 nm thick.

### Electromechanical properties and possible applications

One of the possible applications of the developed composite material is related to flexible electronic components and piezoresistive strain sensors. Among other strain sensor groups, piezoresistive strain sensors have the advantages of simplicity and widely tunable mechanical and electrical properties^29^. Particularly, flexible electronics and miniaturized strain sensors recently gained tremendous interest in wearable and implantable devices for health monitoring and biomedical fields^29–34^.

Highly sensitive piezoresistive sensors usually are made of composite 3D micro/nanostructures often with a carbon material component in the form of graphene nano-flakes infused in rubber-like adhesive pads^29^, carbon nanotube/graphene networks^30^, graphene nanoplatelets or carbon nanotubes with polydimethylsiloxane elastomer^31,32^, graphene/silver nanowire networks^33^, etc.

**Figure 8:**
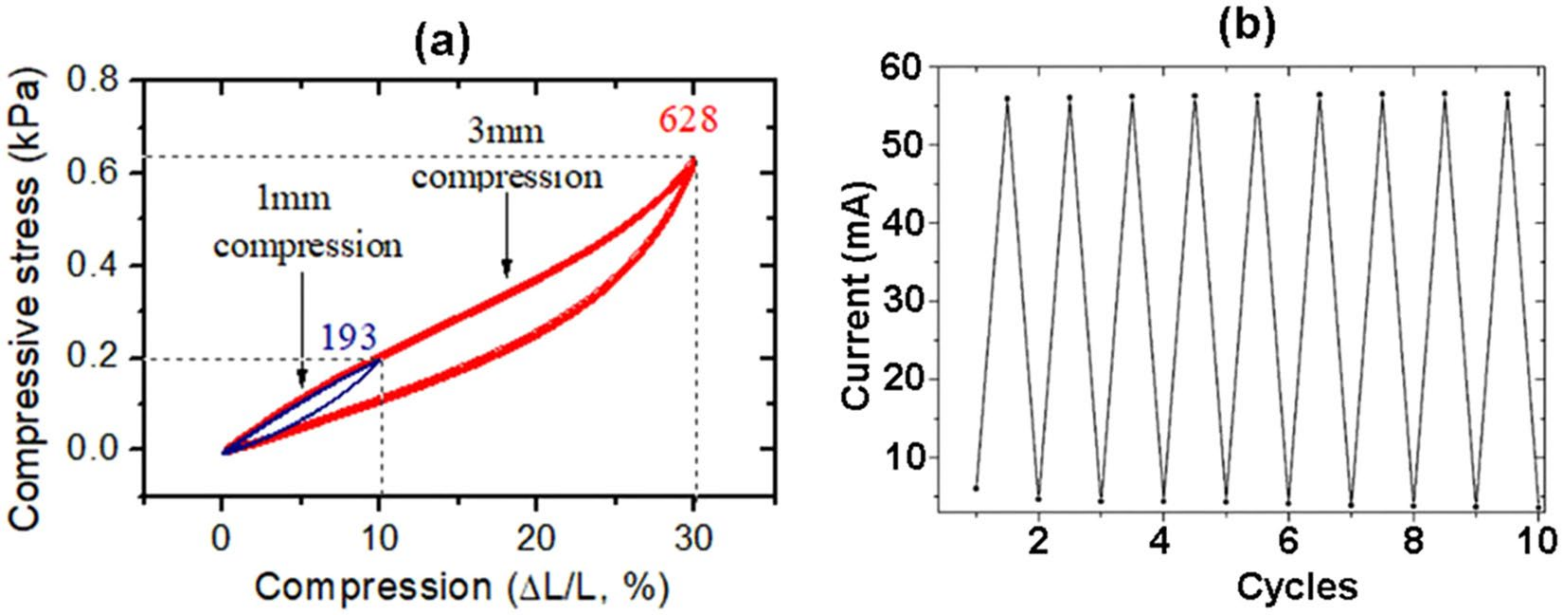
Electromechanical investigations on an Aerographite-InP 3D hybrid network with the morphology illustrated in Fig. 1. (**a**) Cyclic loading-unloading response (compressive) of an AG-InP network under multiple cycles with compression up to 10% and 30%. (**b**) Change in current values (extremes) under loading and unloading cycles of an AG-InP network.

The mechanical, electrical and piezoelectric properties of components determine the flexibility, stretchability and sensitivity of strain sensors. InP is a piezoelectric material with the measured value of the piezoelectric constant *e*_14_ equaling 0.04 C/m^2^ ^35^, and the calculated value of the piezoelectric modulus *d*_14_ equaling −1.8 pC/N^36^. In order to estimate the applicability of the developed AG-InP networks as strain sensors, electromechanical investigations have been performed and the corresponding results are presented in Fig. 8.

The stress-strain behavior of an Aerographite-InP hybrid network subjected to multiple compression cycles demonstrates excellent elastic properties when the compression maxima are set at 10 and 30%, revealing two characteristic deformation regimes: a Hookean nearly linear elastic regime for the compression of up to 25% and a densification regime for higher compressions. The maximum stress was 500 Pa for the linear regime and 628 Pa for the densification regime, at 30% strain. This value is similar to that observed in Aerographite-GaN 3D hybrid networks^3^. However, the thorough analysis under the loading-unloading cycles presented in Fig. S8 (Supplementary Information) demonstrates better elastic properties of the AG-InP network as compared to the AG-GaN one. The AG-GaN hybrid network showed a plastic deformation of 3% at 10 cyclic compressions, while the AG-InP network plastic deformation is less than 1% after the same number of compressions. This highlights the structural robustness of the AG-InP 3D hybrid material. Note that the AG-InP hybrid network shows advantages in comparison with a bare Aerographite network. As one can see from Fig. S9 (Supplementary Information), the compressive stress of the AG-InP hybrid network is higher by a factor of three as compared to that of the bare AG under the same compression of 45%.

The reversible changes in the mechanical properties, especially in piezoelectric materials, may lead to reversible changes in the electrical conductivity, which in turn represent a basis for the development of strain sensors. In a wider context, when one deals with flexible micro- and nano-architectures with a high potential of being incorporated into a variety of device structures, the possibility to change in a controlled fashion the electrical conductivity constitutes a basis for broader applications beyond the strain sensors, actuators, self-reporting materials, etc. To make use of the possibility to modulate the electrical conductivity one needs to have good electrical contacts. The current-voltage characteristics of contacts deposited on the AG-InP network demonstrate a good linearity with insignificant hysteresis due to piezoelectric effects, as shown in Fig. S10 (Supplementary Information).

The dependence of electrical conductivity of an AG-InP network as a function of the compression and compressive stress is shown in Fig. S11 (Supplementary Information), and the stress dependent resistivity behavior over multiple compression cycles is presented in Fig. 8b. Figure S11 demonstrates that the main changes in the electrical resistivity occur in the compression range up to 10%, i.e., in the Hookean elastic regime. Bearing in mind the possible impact of the piezoelectric effect mentioned above, we suggest that the decrease of resistivity under compression is mainly due to the increase in the number of conductive pathways in the AG-InP network structure.

It is known that exposure of bulk InP crystals to air leads to surface oxidation, i.e., to the formation of a native oxide film with a thickness of 2–3 nm. As previously shown^37^, under mechanical compression the ultrathin oxide film is subjected to plastic deformation which, to a certain extent, provides conditions for elastic deformation of single crystalline InP matrix underneath the surface oxide film. In our opinion, good mechanical flexibility and elasticity of the hybrid network along with high transparency of the ultrathin oxide film for free charge carriers ensure stable and rather strong dependence of the electrical conductivity of the AG-InP composite material upon the compression-decompression cycles. Note that the original value of the resistivity is completely recovered upon decompression, as shown in Fig. 8b. It means that no internal damage occurs in the AG-InP network subjected to multiple compression cycles, highlighting better suitability of the developed nanocomposite material for strain sensor applications as compared to AG-GaN hybrid networks reported previously^3^.

## Conclusions

In conclusion, fabrication of three-dimensional flexible networks based on Aerographite decorated by InP micro/nanocrystallites is demonstrated by using HVPE techniques. The growth of zincblende InP micro/nanocrystallites is disclosed on both outer and inner surfaces of the graphitic microtubular structures, the InP micro/nanostructures being uniformly distributed and strongly attached to the surface of the graphite walls thus preventing their agglomeration. Electromechanical studies reveal high elasticity and controlled modulation of the electrical conductivity of the hybrid composite material under compression/decompression cycles. The stress dependent electrical conductivity of the AG-InP hybrid networks may find applications in tactile/strain sensors and other device structures related to flexible electronics. Functionalization of the Aerographite backbone by Au dots is found to drastically change the mechanisms of InP growth which leads to the formation of InP micrometer-scale arrow-shaped rods, In_2_O_3_ nanowires and core-shell nanostructures with gold core and indium oxide shell, the generation of indium oxide being caused by the fast oxidation in air of the nanoscale InP structures.

## Materials and Methods

### Synthesis of Aerographite 3D Templates

The Aerographite networks are produced by a one-step chemical vapor deposition (CVD) process described elsewhere^38^. Highly porous ZnO networks with a 3D architecture, which are entirely built-up from interconnected micrometer thick rods, often in the shape of tetrapods and multipods, are used as sacrificial templates^39^. These sacrificial templates are converted to graphite networks by the CVD process, which incorporates a heat treatment at 760 °C in an argon/hydrogen atmosphere. Evaporated toluene provides carbon for the nucleation and growth of enclosing graphitic shells, whilst the underlying ZnO network is reduced and removed by the gas flow. Since the conversion process exactly follows the template structure, the final Aerographite material is almost exact mimicry from sacrificial ZnO template architecture in which ZnO has been replaced by carbon in the form of tubular graphitic carbon^2^.

### Growth of Aerographite-InP 3D Networks

The HVPE growth process was carried out in a chamber with three heat zones at a constant hydrogen flow, under atmosphere pressure. It includes the following steps: the purification of the metallic In source by thermal treatment at 750 °C in the flow of Pd filtered H_2_, saturation of the In source with phosphorus in the flow of PCl_3_-H_2_ at 750 °C and finally growth of Zn doped p-InP on the Aerographite substrate at 650 °C from InP-Zn-H_2_ vapors. The first heat zone is maintained at 750 °C to generate In vapors from the In precursor. In the second zone, maintained at 720 °C, the In vapors react with PCl_3_. The constant flow of H_2_ transports the gaseous phases to the third zone, where they crystallize on the substrate at the temperature of 650 °C. For p-type doping, a Zn precursor is evaporated at 200 °C in a separate chamber and is introduced into the third zone.

To investigate the influence of Au catalyst on the growth of InP microcrystals, a gold film with a thickness of 2.5/5/7/13 nm was deposited on the AG scaffold in a Cresington sputtering system. Subsequently, the gold films were annealed at 400 °C for 1 h in normal conditions, this process resulting in the formation of gold nanoparticles.

### Characterization

The microstructure evolution of pure Aerographite and AG-InP hybrid networks was investigated by scanning electron microscopy on a Zeiss Ultra Plus and a VEGA TESCAN TS 5130MM. The chemical composition analysis of AG-InP networks was carried out with the EDX accessory of the SEM instrument. X-ray diffraction studies were performed by using a 3000 PTS Seifert machine (with Cu Kα radiation with a wavelength of 0.15418 nm, 2500 Watt X-ray tube operated at 50 kV and 40 mA). Data were collected by step counting at 0.05 deg. intervals for 2 seconds per data point.

Transmission electron microscopy analysis was performed with a Tecnai F30 STwin electron microscope (300 kV, field-emission gun, spherical aberration constant Cs 1.2 mm). The EDX analysis was also performed in the TEM mode with a Si/Li detector.

### Photoluminescence Measurements

Photoluminescence (PL) measurements were performed using a Bruker Vertex 80 v Fourier Transform Infrared (FTIR) spectrometer equipped with an InGaAs detector. The sample was inserted in a helium flux cryostat that allowed changing the temperature from 4.5 to 300 K. A 457.9 nm laser with the maximum power of 166 mW was used as excitation source.

### Raman Measurements

Raman spectra were measured in the backscattering geometry at room temperature using a Jobin Yvon micro-Raman spectrometer HR800 with 633 nm or 532 nm laser excitation via a 50× microscope objective. In order to avoid the heating of the samples by the laser, low laser powers below 0.6 mW were used for both laser excitations. However, to test the laser heating influence on the Raman spectra, also larger laser powers were used (see Supplementary Information).

### Electromechanical Measurements

The electro-mechanical measurements were conducted with a self-designed computer controlled setup, which consists of a Kern PLE 310-3 N precision balance and a Märzhauser Wetzlar HS 6-3 micromanipulator. The setup allows a stepwise tensile or compressive deformation of the sample up to an arbitrary number of cycles while the force is measured by the balance. The setup also allows the simultaneous measurement of the electrical resistance of the sample for each deformation step by a Keithley 2400 source meter^3,40^, which is connected to the sample by gold plated copper contacts.

## Electronic supplementary material

Supplementary Information
