# Supplementary material for: Hierarchical Aerographite 3D flexible networks hybridized by InP micro/nanostructures for strain sensor applications
Source: Sci Rep. 2018 Sep 17;8:13880. doi: 10.1038/s41598-018-32005-0 (PMC6141564; doi:10.1038/s41598-018-32005-0)
Supplement: Supplementary file 1 — Supplementary Information [file 41598_2018_32005_MOESM1_ESM.docx]

**SUPPLEMENTARY INFORMATION**

**Hierarchical Aerographite 3D flexible networks hybridized by InP micro/nanostructures for strain sensor applications**

Irina Plesco^1^, Julian Strobel^2^, Fabian Schütt^2^, Cameliu Himcinschi^3^, Nabiha Ben Sedrine^4^, Teresa Monteiro^4^, Maria Rosário Correia^4^, Leonid Gorceac^5^, Boris Cinic^5^, Veaceslav Ursaki^1^, **Janik Marx**^6^**,** Bodo Fiedler^6^, Yogendra Kumar Mishra^2^, Lorenz Kienle^2*^, Rainer Adelung^2*^& Ion Tiginyanu^1*^

*^1^ National Center for Materials Study and Testing, Technical University of Moldova, Stefan cel Mare av. 168, MD-2004 Chisinau, Republic of Moldova*

*^2^ Institute for Materials Science, Kiel University, Kaiserstr. 2, D-24143 Kiel, Germany*

*^3^ Institute of Theoretical Physics, TU Bergakademie Freiberg, Leipziger Str. 23, D-09596 Freiberg, Germany*

*^4^ Department of Physics and I3N, Institute for Nanostructures, Nanomodelling and Nanofabrication, University of Aveiro, P-3810-193 Aveiro, Portugal*

*^5^ Department of Physics and Engineering, State University of Moldova, Alexei Mateevici str. 60, MD-2009 Chisinau, Republic of Moldova*

*^6^ Institute of Polymers and Composites, Hamburg University of Technology, Denickestr. 15, D-21073, Hamburg, Germany*

**
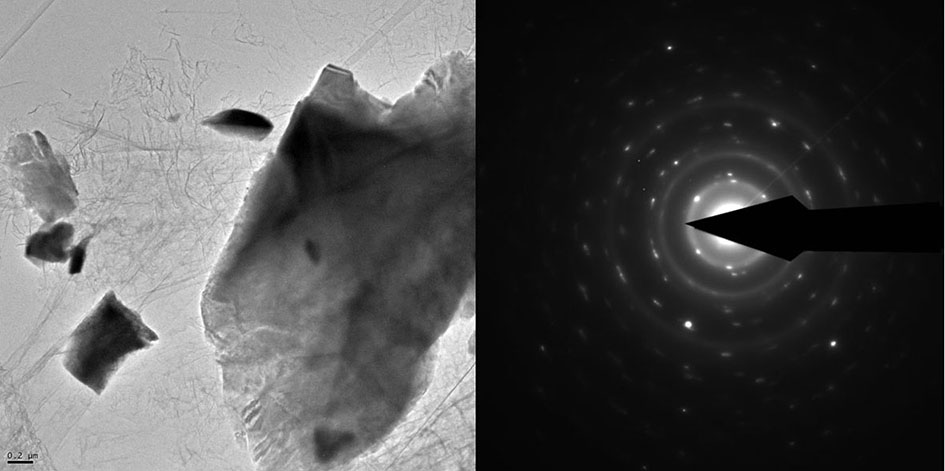
**

**Figure S1**. Overview image and SAED of an InP microcrystal. The diffraction pattern shows single crystalline InP (sg. F$\bar{4}3$m) and some polycrystalline rings from the AG scaffold.

**
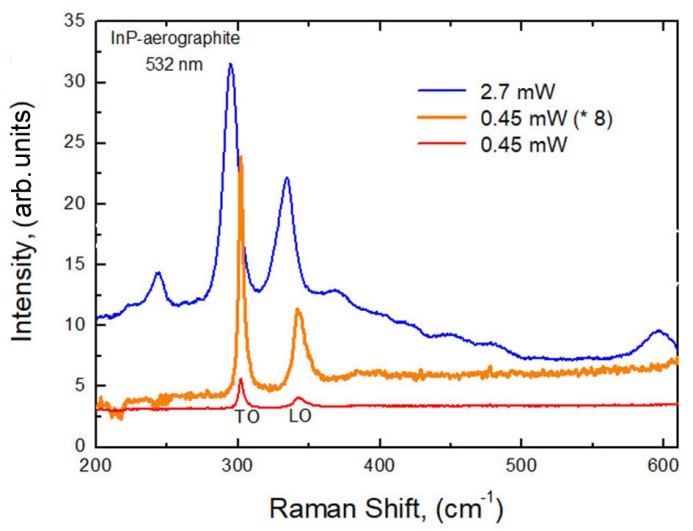
**

**Figure S2.** Raman scattering spectra of InP crystallites deposited on Aerographite, measured at room temperature under excitation with the wavelength of 532 nm for two excitation laser powers of 0.45 mW and 2.7 mW.

**
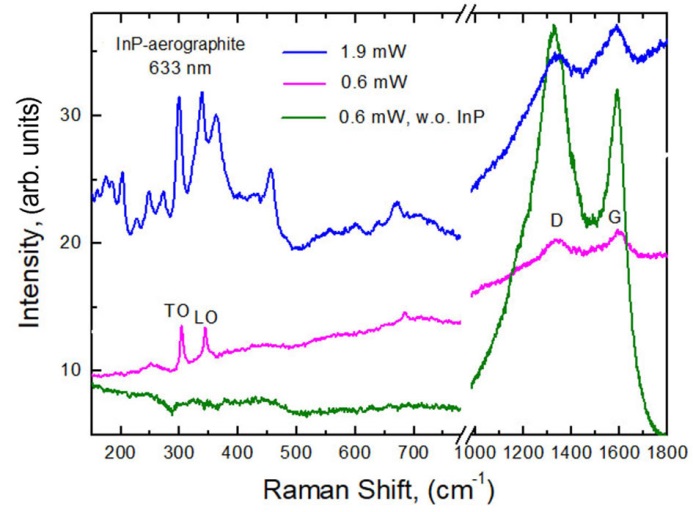
**

**Figure S3.** Raman scattering spectra of InP crystallites deposited on Aerographite, measured at room temperature under excitation with the wavelength of 633 nm at two excitation laser powers of 0.6 mW and 1.9 mW. For the purpose of comparison, the Raman spectrum from the virgin Aerographite (without InP) is shown.


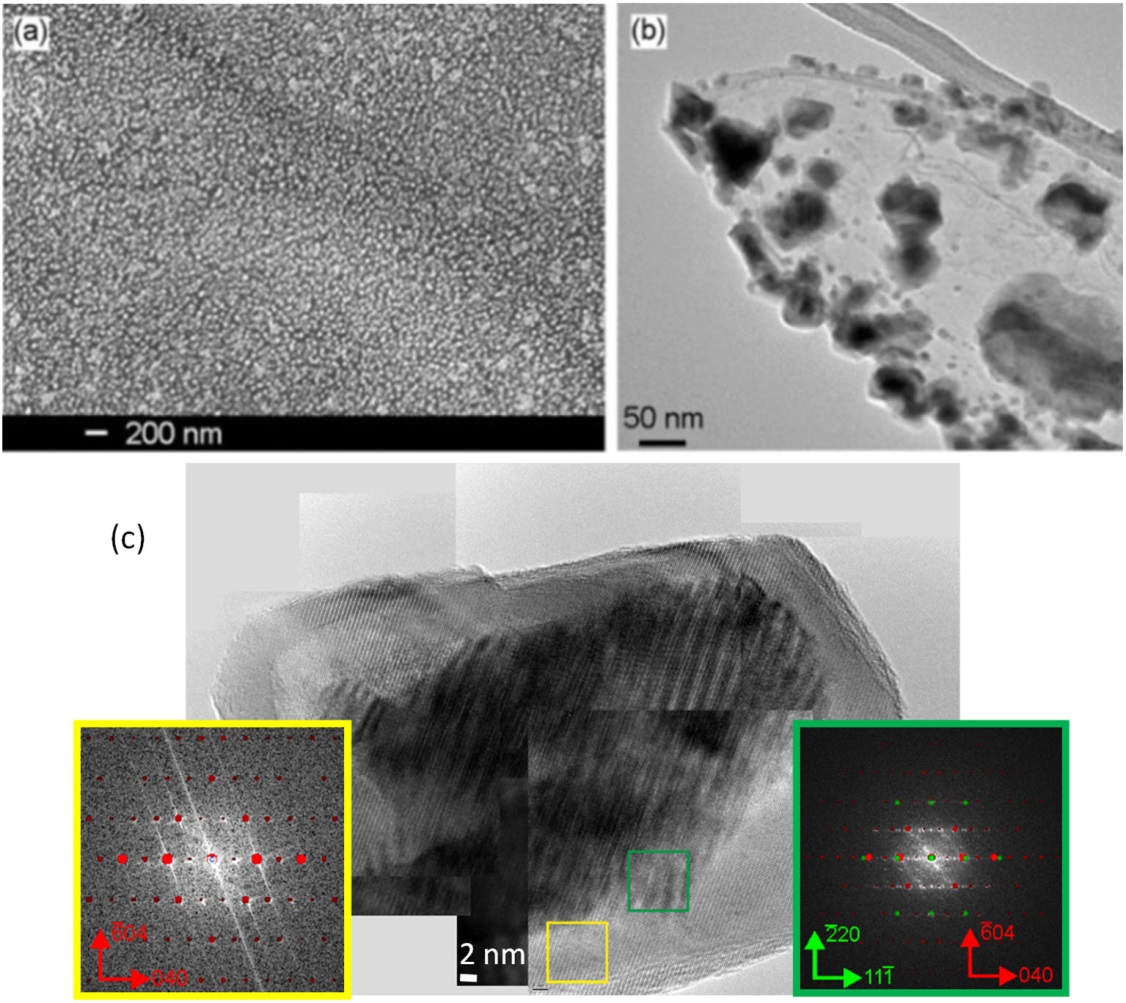


**Figure S4.** (a) SEM image of Au nanoparticles deposited on the AG template. (b) TEM image of an AG microtube with nanoparticles deposited at the initial phase of the HVPE process. (c) Composed HRTEM image of a deposited nanoparticle proving the epitaxially intergrown Au core with In_2_O_3_ shell. Inset is the FFT of the core and the shell regions.


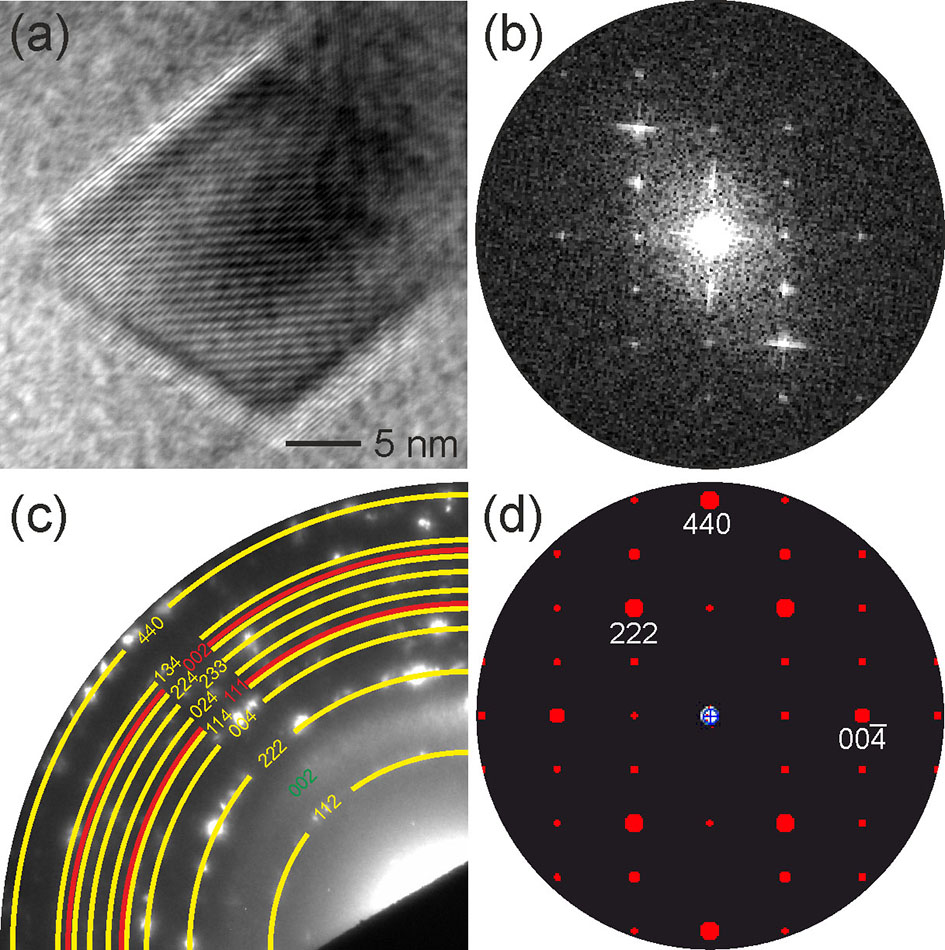


**Figure S5.** TEM analysis of In_2_O_3_ particles. (a) High resolution micrograph of a single particle and (b) FFT of this particle. (c) SAED pattern of an area covered with particles, containing In_2_O_3_ (yellow), Au (red) and graphite (green) diffraction maxima. (d) Simulated diffraction pattern of In_2_O_3_ (ZA [1-10]) matching perfectly with the FFT in (b). Some indices for the respective diffraction spots are given.


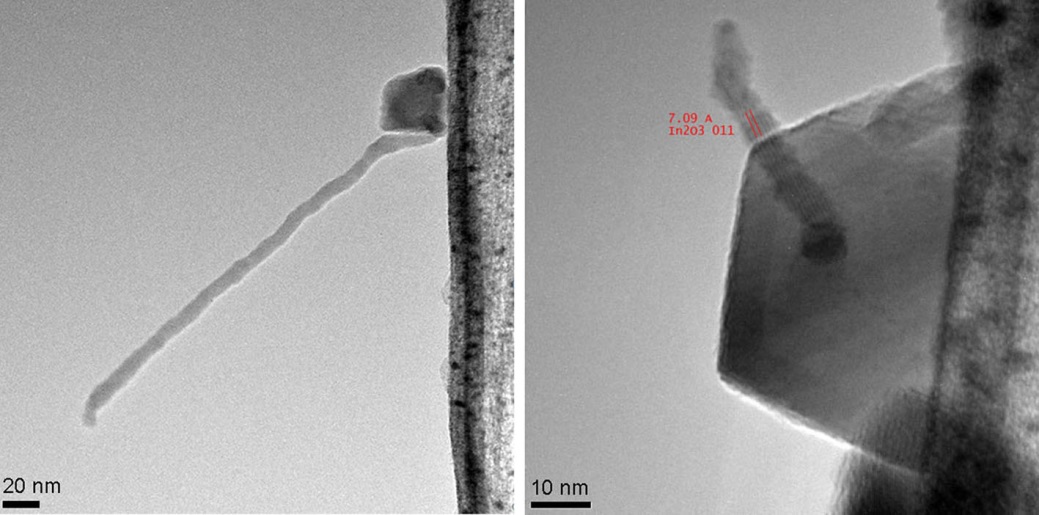


**Figure S6**. HRTEM image of a nanoparticle around 40 nm large attached to the AG template and a nanowire with the length of 250 nm grown from the nanoparticle. The nanowire is crystalline and shows the same In_2_O_3_ lattice spacing as the shell of the nanoparticle.


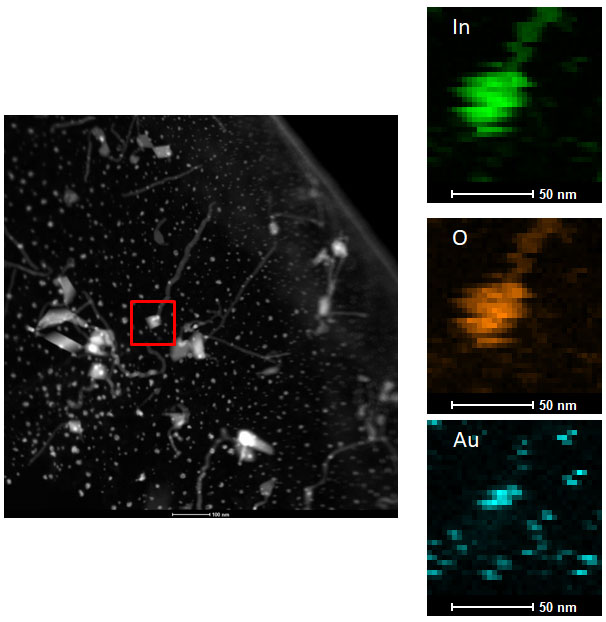


**Figure S7**. EDX mapping of a nanoparticle and a nanowire grown with Au catalyst on the Aerographite showing the Au/In_2_O_3_ core-shell character. Some artifacts from pronounced sample drift and improper drift correction are apparent.


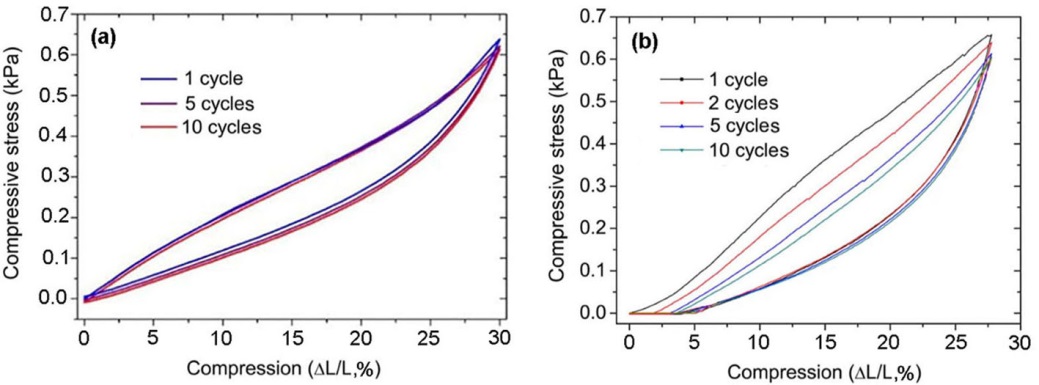


**Figure S8.** A comparison of cyclic loading-unloading response (compressive) of the AG-InP (a) and AG-GaN (b) networks under multiple cycles with compression up to 30 %.


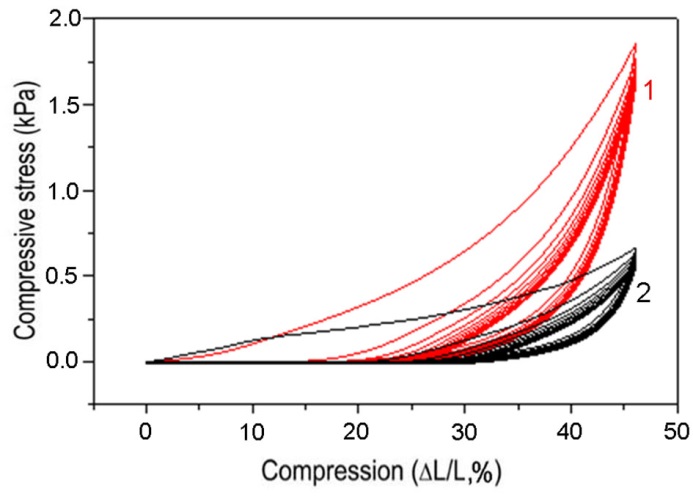


**Figure S9.** A comparison of cyclic loading-unloading response (compressive) of the AG-InP (red curves) and bare AG (black curves) networks under multiple cycles with compression up to 45 %.


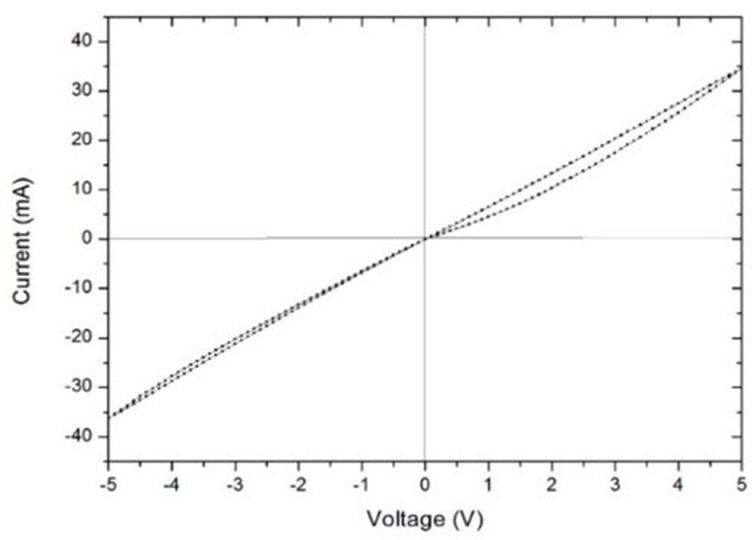


**Figure S10.** Current-voltage response of an AG-InP network.


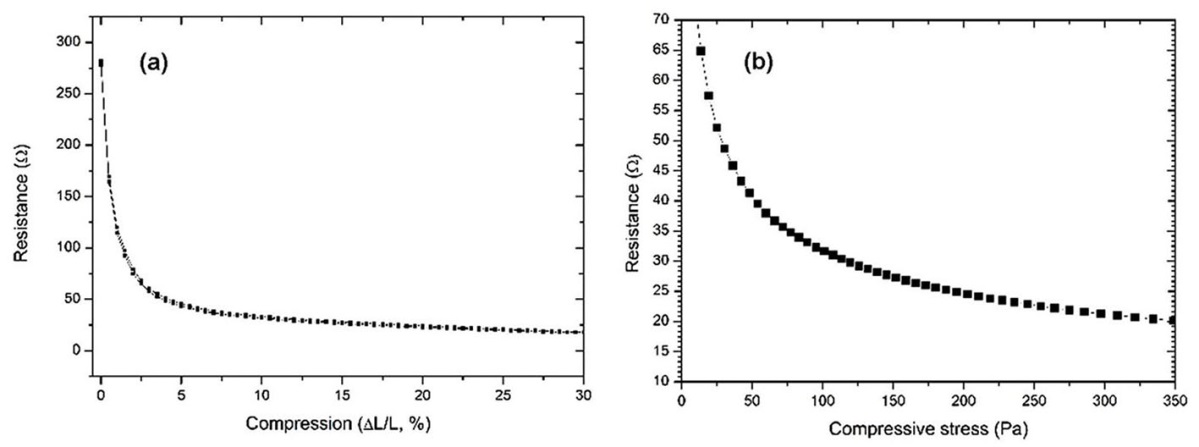


**Figure S11.** The decrease in electrical resistivity of an AG-InP network as a function of compression (a) and compressive stress (b).
